# Chlamydia trachomatis TmeA Directly Activates N-WASP To Promote Actin Polymerization and Functions Synergistically with TarP during Invasion

**DOI:** 10.1128/mBio.02861-20

**Published:** 2021-01-19

**Authors:** Gabrielle Keb, Joshua Ferrell, Kaylyn R. Scanlon, Travis J. Jewett, Kenneth A. Fields

**Affiliations:** a Department of Microbiology, Immunology, and Molecular Genetics, University of Kentucky College of Medicine, Lexington, Kentucky, USA; b Division of Immunity and Pathogenesis, Burnett School of Biomedical Sciences, College of Medicine, University of Central Florida, Orlando, Florida, USA; Yale University School of Medicine

**Keywords:** type III secretion, FRAEM, FLAEM, N-WASP

## Abstract

Chlamydia trachomatis is a medically significant human pathogen and is an epithelial-tropic obligate intracellular parasite. Invasion of nonprofessional phagocytes represents a crucial step in the infection process and has likely promoted the evolution of a redundant mechanism and routes of entry. Like many other viral and invasive bacterial pathogens, manipulation of the host cell cytoskeleton represents a focal point in *Chlamydia* entry. The advent of genetic techniques in C. trachomatis, such as creation of complete gene deletions via fluorescence-reported allelic exchange mutagenesis (FRAEM), is providing important tools to unravel the contributions of bacterial factors in these complex pathways. The type III secretion chaperone Slc1 directs delivery of at least four effectors during the invasion process. Two of these, TarP and TmeA, have been associated with manipulation of actin networks and are essential for normal levels of invasion. The functions of TarP are well established, whereas TmeA is less well characterized. We leverage chlamydial genetics and proximity labeling here to provide evidence that TmeA directly targets host N-WASP to promote Arp2/3-dependent actin polymerization. Our work also shows that TmeA and TarP influence separate, yet synergistic pathways to accomplish chlamydial entry. These data further support an appreciation that a pathogen, confined by a reductionist genome, retains the ability to commit considerable resources to accomplish bottle-neck steps during the infection process.

## Abstract (precis)

The increasing genetic tractability of Chlamydia trachomatis is accelerating the ability to characterize the unique infection biology of this obligate intracellular parasite. These efforts are leading to a greater understanding of the molecular events associated with key virulence requirements.

## INTRODUCTION

*Chlamydia* is an obligate intracellular bacterial pathogen that includes C. trachomatis, C. pneumoniae, and C. psittaci, all of which are capable of mediating a range of infections in humans (1). C. trachomatis is the most common bacterial sexually transmitted infection in the United States (2) and is also a leading cause of blindness, affecting nearly 2.2 million people worldwide with ocular trachoma (3). C. pneumoniae causes infection of the upper respiratory tract and is responsible for 10 to 20% of adult community-acquired pneumonia (4), whereas C. psittaci represents a zoonotic species, with accidental infection of animal handlers being most common (5).

All *Chlamydia* spp. manifest a hallmark biphasic developmental cycle consisting of infectious elementary body (EB) and noninfectious, reticulate body (RB) forms. Development occurs entirely within a parasitophorous vacuole termed an inclusion. *Chlamydia* preferentially infects columnar epithelial cells and appears to utilize multiple mechanisms for entry into these nonphagocytic cells (6–10). Actin is rapidly recruited to the site of chlamydial attachment (9), and manipulation of the host cytoskeleton plays a central and pivotal role during the invasion process. *In vitro* studies have shown that invasion of C. trachomatis is significantly impaired when actin polymerization or depolymerization is disrupted with either cytochalasin D or jasplakinolide, respectively (reviewed in reference 11). It has been well established that central host factors responsible for manipulating actin dynamics, including the RAS-related C3 botulinum toxin substrate 1 (Rac1), Wiskott-Aldrich syndrome protein family member 2 (WAVE2), and actin-related proteins 2 and 3 (ARP2/3), play important roles during C. trachomatis internalization (reviewed in reference 11). Beyond invasion, the actin-based cytoskeleton is involved in maintaining inclusion integrity during intracellular development (12) and host cell exit by exocytosis (13).

*Chlamydia* initially adheres to the host plasma membranes via low-affinity interactions such as those manifested between heparan sulfate proteoglycans and C. trachomatis outer membrane proteins, such as OmcB (14). Subsequent high-affinity interactions are established between chlamydial outer membrane proteins and host receptors, such as integrin β1 (ITGβ1) (15), epidermal growth factor receptor (EGFR) (16), ephrin receptor A2 (EPHA2) (6), or platelet-derived growth factor receptor β (PDGFRβ) (17). These high-affinity interactions can mediate chlamydial attachment leading to receptor clustering and downstream actin remodeling, culminating in invasion (6–10). For example, C. trachomatis Ctad1 binds to ITGβ1 and induces receptor clustering, activation of Erk1/2, and chlamydial internalization (15). The individual activities of both EGFR (18) and EPHA2 (6) also contribute to chlamydial entry. These receptors presumably represent individual, redundant doorways for *Chlamydia* to cross the host’s plasma membrane barrier.

In addition to receptor-mediated endocytosis, actin-containing filopodia that form distinct cup, tail, and ruffle structures have been noted during chlamydial invasion (9, 19). Detailed structural and biochemical analyses have recently revealed that these structures correspond to events associated with macropinocytosis-mediated entry and have implicated novel contributions of the Bin/amphiphysin/Rvs (BAR) domain protein sorting nexin 9 (SNX9), cell division control protein 42 (Cdc42), and neural Wiskott-Aldrich syndrome protein (N-WASP) during chlamydial infection. Macropinocytosis is an actin-dependent process where extended filopodia fuse with the plasma membrane to form fluid-phase endocytic compartments termed macropinosomes (20). SNX9 contributes to membrane curvature and can impact actin dynamics by recruiting Cdc42 and N-WASP (21). All three host proteins are recruited to EB attachment sites, and infection of SNX9^−/−^ cells or pharmacologic inhibition of Cdc42 or N-WASP negatively impacts invasion (19). These data indicate that *Chlamydia* may deploy effector proteins that manipulate macropinocytosis to effect entry. In support of this notion, C. pneumoniae Cpn0678 binds directly to SNX9 to effect membrane curvature associated with entry events (22).

C. trachomatis employs a type III secretion system (T3SS) to inject anti-host effector proteins into associated cells (23). The translocated actin-recruiting phosphoprotein (TarP) was the first effector discovered that is deployed during invasion (8) and is now well established as a factor that spatially and temporally recruits actin to the site of EB invasion (reviewed in reference 11). TarP is a multidomain protein containing an N-terminal repeat domain containing tyrosine residues, a proline-rich domain, one G-actin binding domain, two C-terminal F-actin binding domains (24), and domains impacting the dynamics of focal adhesions (25). Once secreted, TarP is immediately phosphorylated at tyrosine residues by host tyrosine kinases and can directly bind Sos1 and Vav2, and Rac1 guanine nucleotide exchange factors (GEFs), to stimulate Rac1-dependent signaling for actin recruitment (8, 26). Independent of phosphorylation, TarP can directly nucleate or bundle actin through its G-actin and F-actin domains, respectively (24, 27). C. trachomatis strains containing *tarp* gene deletions are significantly inhibited during invasion, thus highlighting TarP as a critical invasion-related effector (28).

TarP is one of at least four Slc1-chaperoned effectors secreted during invasion. This family also includes translocated early phosphoprotein (TepP) and translocated membrane-associated effectors A and B (TmeA and TmeB). TepP is phosphorylated after secretion, yet later than TarP (29). Through interactions with CrkI-II, TepP is associated with regulation of innate immune responses during infection (29). In other cell types, Crk activation has been linked to Rac1-dependent cytoskeletal reorganization, where ITGβ1 activation leads to Crk docking at the plasma membrane with DOCK180, a Rac1-specific activator (30, 31). TmeA is also implicated in actin reorganization (32, 33). TmeA localizes to host plasma membranes (32) and has been shown to disrupt the actin bundling activity of AHNAK, a large host scaffolding protein (34). A C. trachomatis strain harboring a *tmeA* gene deletion is defective for invasion (34) and displays a phenotype similar to a *tarp* null strain (28). The interaction with AHNAK does not correlate with the invasion defect manifested by *tmeA*-depleted strains, leaving the molecular mechanism governing TmeA activity an open question. Finally, TmeB localizes with the inclusion and plasma membranes (35); however, its function has yet to be elucidated.

It is clear that the Slc1-chaperoned effectors play a role in actin rearrangements during chlamydial invasion, with the functions of TarP and TmeA being most prominent. The high degree of redundancy among actin reorganization pathways has made it difficult to identify specific roles and the collective impact of T3S effectors during chlamydial invasion. We have developed two approaches for generating compete gene deletions in C. trachomatis L2. In this study, we leveraged fluorescence-reported allelic exchange mutagenesis (FRAEM) (36) and floxed-cassette allelic exchange mutagenesis (FLAEM) (37) to generate a single strain harboring complete deletions of both *tarp* and *tmeA*. With this double mutant strain, in combination with single mutant strains and biochemical approaches, we provide evidence that these effectors impact separate signaling pathways that converge at Arp2/3. Furthermore, we show evidence that TmeA can position proximal to receptors implicated in chlamydial entry. TmeA is required for focusing of N-WASP at sites of entry, binds to N-WASP, and is sufficient to directly activate N-WASP-dependent Arp2/3 activity to stimulate actin polymerization. These data indicate that TarP and TmeA act synergistically to promote the invasion of host cells by C. trachomatis.

## RESULTS

**FIG 1:**
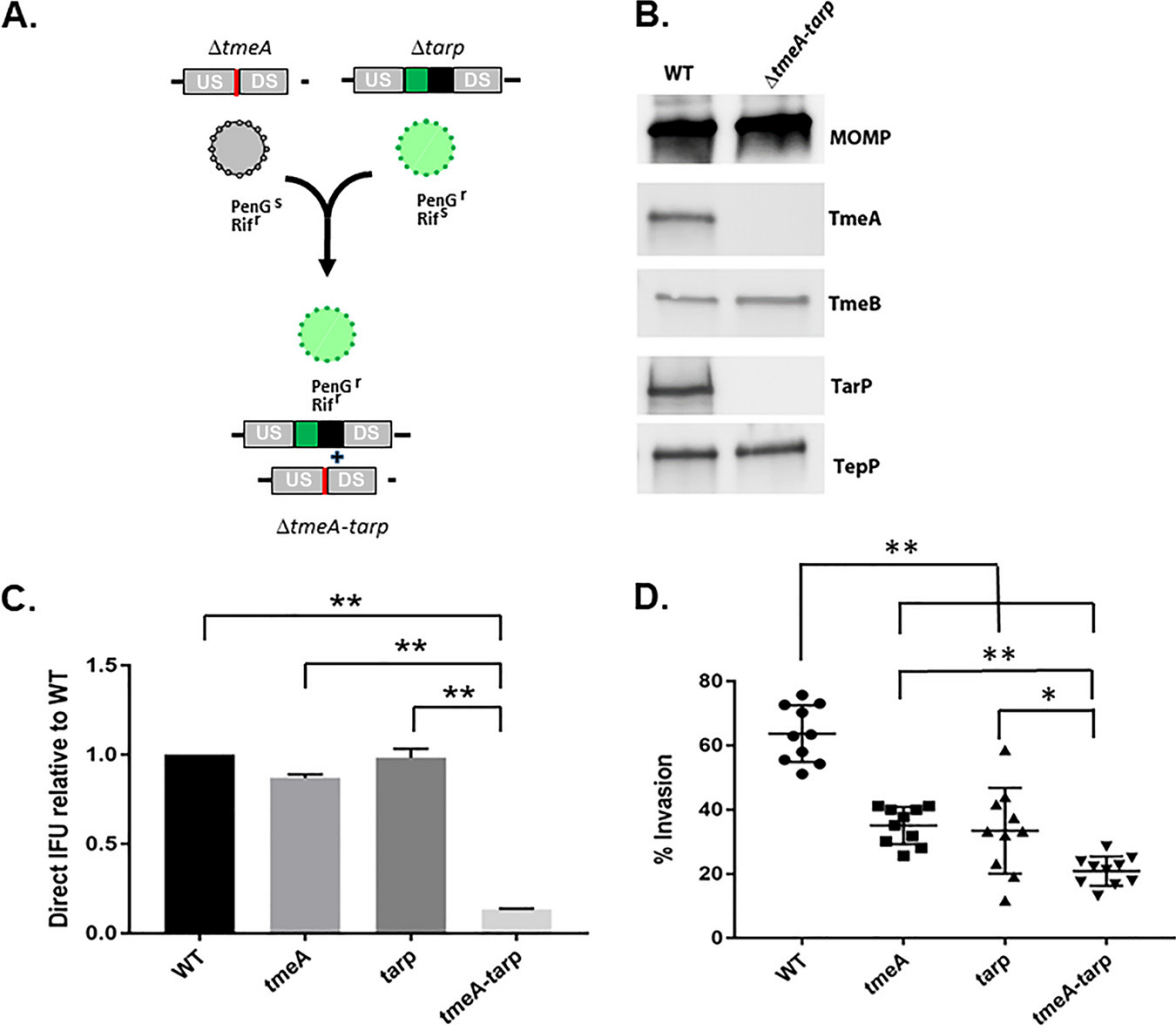
C. trachomatis lacking both *tmeA* and *tarp* is more severely attenuated for invasion. (A). Schematic for application of lateral gene transfer to generate Δ*tmeA-tarp* from single mutant strains. Rifampin-resistant (Rif^r^), penicillin-sensitive (PenG^s^) Δ*tmeA* lacking GFP was cocultured with PenG^r^, Rif^s^ Δ*tarp* to allow lateral gene transfer between strains. Both PenG and Rif selection were applied to isolate a GFP^+^ strain lacking *tmeA* and *tarp*. (B). Immunoblot analysis of material from DG-purified wild-type (WT) or double mutant (Δ*tmeA-tarp*) EBs. SDS-PAGE-resolved material was probed with antibodies specific for effectors TmeA, TmeB, TarP, and TepP or MOMP as a loading control. (C). HeLa cells were infected using equivalent numbers of WT or *tmeA*-, *tarp*-, or *tmeA-tarp*-deficient strains at an approximate MOI of 0.1. At 24 h postinfection, cultures were methanol fixed and stained to enumerate chlamydial inclusions. WT inclusion numbers were set to 1.0, and data from mutant strains are represented as the mean ± standard deviation of triplicate samples. (D). HeLa monolayers were infected for 1 h at 4°C with WT or mutant strains at an MOI of 10. Cultures were shifted to 37°C for 30 min and then paraformaldehyde fixed and processed for inside-out staining to assess invasion efficiency. Data are represented as mean values for percentage of internalized chlamydiae and are shown with standard deviations. Statistical significance was computed using Student’s *t* test with Welch’s correction (*, *P* < 0.002; **, *P* < 0.0001).

Our mutagenesis studies have shown that TmeA (34) and TarP (28) are individually required for efficient invasion of epithelial cells. Strains lacking either effector manifest a modest ca. 40 to 50% defect in invasion compared to the wild type (WT). Since TarP and TmeA have both been implicated in manipulation of the host cytoskeleton, we wondered if they might function synergistically to promote invasion. A C. trachomatis strain lacking both *tmeA* and *tarp* was created to address this question. We leveraged the markerless deletion of *tmeA* in combination with the comparative efficiency of lateral gene transfer to generate the double mutant from existing *tarp* and *tmeA* strains. McCoy cells were coinfected (Fig. 1A) with Δ*tmeA* expressing rifampin (Rif) resistance and Δ*tarp* expressing resistance to penicillin G (PenG). Cultures were serially passaged in the absence of selection, and the double mutant was subsequently isolated by cultivation in the presence of both Rif and PenG. Isolation of a clonal strain resulted in a single strain deficient in both *tmeA* and *tarp*. Gene deletion was confirmed by sequencing and immunoblot analysis. TmeA and TarP-specific signals were absent from EBs, whereas the additional Slc1-chaperoned effectors, TmeB and TepP, were still detected (Fig. 1B). Enumeration of inclusion-forming units (IFUs) after particle-normalized infections has been used successfully as an indicator of defects during early infection events (34). The fitness of the double mutant was compared to single mutants and the WT by infecting HeLa cells with equal numbers of EBs (Fig. 1C). Assessment of mature inclusions at 24 h postinfection revealed that Δ*tmeA-tarp* was significantly attenuated compared to WT and single mutant strains. We then compared the invasion efficiency of the double mutant with single mutant strains and WT bacteria (Fig. 1D). Infections were synchronized by attachment at 4°C and then shifted to 37°C for 30 min prior to fixation and differential staining of extracellular and intracellular bacteria; 63.7% (± 8.8%) of WT EBs were intracellular by 30 min. Consistent with previous reports, invasion efficiency was decreased ca. 50% in single mutant strains to 35% (± 5.7%) for Δ*tmeA* and 33.5% (± 13.3%) for Δ*tarp*. In the absence of both TmeA and TarP, invasion was further decreased to 20% (± 4.5%). These data indicate that TmeA and TarP function in an additive fashion to promote efficient uptake of *Chlamydia*.

TmeB and TepP were expressed in the double mutant (Fig. 1B), and neither have been previously implicated in the invasion process. We examined translocation of TepP by tyrosine phosphorylation (29) and TmeB by subcellular fractionation (32) to exclude the possibility that the invasion phenotype manifested in our single and double null strains depends on altered secretion of TepP and/or TmeB (Fig. S1). TepP phosphorylation was readily apparent in all strains (Fig. S1A). Likewise, TmeB secretion was also apparent, since Triton-X114 extractions revealed a transition of TmeB from soluble to membrane-containing fractions (Fig. S1B). Finally, we directly ruled out potential roles of TepP and TmeB in decreased invasion by testing entry efficiency of strains lacking either effector. The absence of neither *tepp* nor *tmeB* negatively impacted invasion efficiency (Fig. S1C). Therefore, the decreased invasion efficiency manifested by the Δ*tmeA-tarp* strain is likely not mediated by alterations in TmeB or TepP secretion.

10.1128/mBio.02861-20.2 FIG S1 Loss of TmeA and Tarp does not indirectly impact invasion via effects on TepP or TmeB. (A). Tyrosine phosphorylation of TarP and TepP was assessed in whole-culture material harvested 45 min after infections with equivalent IFUs of WT or strains lacking, *tarp*, *tmeA*, *tmeA*, and *tarp* or *tepp*. SDS-PAGE-resolved material was probed with anti-phosphotyrosine-specific antibodies to visualize phosphor-TarP and TepP, while tubulin and MOMP were detected as loading controls for host and chlamydial material, respectively. (B). Pure WT EBs or HeLa cell monolayers infected with WT and mutant strains at an MOI of 100 for 1 h were subjected to Triton X-114 detergent extraction. Proteins in detergent (Det) and aqueous (Aq) phases were concentrated, and fractions were probed in immunoblot analysis with TmeB- and TepP-specific antibodies. Material was probed for Scc2 and MOMP as aqueous and detergent controls, respectively. (C). HeLa monolayers were infected for 1 h at 4°C with WT or strains lacking *tmeB* or *tepp* at an MOI of 10. Cultures were shifted to 37°C for 30 min and then paraformaldehyde fixed and processed for inside-out staining to assess invasion efficiency. Data are represented as mean values for the percentage of internalized chlamydiae with standard deviations. No statistically significant differences were noted with Student’s *t* test with Welch’s correction. Download FIG S1, TIF file, 0.3 MB. Copyright © 2021 Keb et al. 2021 Keb et al. This content is distributed under the terms of the Creative Commons Attribution 4.0 International license.

Our previous analysis of the TmeA-AHNAK (34) interaction clearly indicated that interactions with other host factors likely contributed to the overt role of TmeA in promoting invasion. Identification of additional TmeA binding partners in host cells was investigated to address the contribution(s) of TmeA during invasion. Proximity labeling has emerged as an efficacious approach for delineating potential interacting partners of chlamydial effectors (34, 38–40). We reasoned that ectopic expression of a TmeA-containing chimeric protein in HeLa cells would provide a nonbiased indication of potentially interacting host proteins. TmeA was fused to the promiscuous biotin ligase, BirA. A similar fusion was created using TmeA lacking the membrane-localization domain (MLD) as a control. Duplicate experiments were performed where HeLa cells were transiently transfected via nucleofection with BirA only, TmeA-BirA, or TmeAΔmld-BirA and cultured in the presence of exogenous biotin for 24 h. A portion of each sample was resolved via SDS-PAGE followed by probing with streptavidin-horseradish peroxidase (HRP) in immunoblots to confirm ligase activity (Fig. S2A) . Nucleofection efficiency and localization of TmeA-containing fusion proteins were confirmed by parallel staining of fixed cells with c-*myc* antibodies (Fig. S2B).

10.1128/mBio.02861-20.3 FIG S2 Functional assessment of BirA-containing chimeras indicates active ligase activity and TmeA-dependent localization in HeLa cells. HeLa cells expressing the promiscuous biotin ligase BirA fused to TmeA or TmeAΔMLD were cultured with and without Biotin. (A) Whole-culture proteins were assayed by immunoblot in conjunction with mock-treated HeLa cells or cells expressing BirA only as a control. Expression of the respective construct was confirmed by probing with α-myc, and actin expression was used as a loading control. Probing with NeutrAvidin-HRP was used to detect biotinylated HeLa proteins. (B) Cells cultured on coverslips for 24 h were fixed with 4% paraformaldehyde (PFA), permeabilized with 0.4% TRITON X-100, and blocked in 5% bovine serum albumin (BSA) Tris-buffered saline with Tween 20 (TBST) for 1 h. Cells were probed with α-myc IgG (green) to detect BirA-TmeA fusions and with NeutrAvidin-Texas red (red) to detect biotin or with DAPI (blue) to indicate host cell nuclei. Confocal images are shown. Bar = 10 µm. Download FIG S2, TIF file, 0.5 MB. Copyright © 2021 Keb et al. 2021 Keb et al. This content is distributed under the terms of the Creative Commons Attribution 4.0 International license.

Common contaminants such as keratin and heat shock proteins, in addition to endogenously biotinylated proteins, such as carboxylases, were excluded from mass spectroscopy (MS) results. We also excluded proteins with a confidence score below 30, thereby setting an established threshold of a 0.001 probability of peptide identification being random. A complete list of identified proteins appears in Table S1. Data were screened for proteins that appeared in the TmeA-BirA samples but not in those from BirA and TmeAΔmld. A total of 12 unique host proteins were detected in the presence of full-length TmeA-BirA (Table 1). As expected, these included the TmeA-interacting proteins AHNAK and AHNAK2. We also detected host proteins previously implicated during *Chlamydia* attachment or invasion, including ITGβ1 (15), *WASL*/N-WASP (19), EphA2 (6), and EGFR (16). Additional proteins included the amino acid transporters (SLC3A2 (CD98hc), SLC7A5 (LAT1), and SLC1A5 (ASCT2), cytoskeleton-associated factors) formin BP1 (FNBP1) and podocalyxin-like protein (PODXL), and the surface receptor CD44.

**TABLE 1:** MS identification of host proteins uniquely proximal to TmeA-BirA

Uniprot accession no.	Description^*a*^	Score^*b*^	Coverage^*c*^	Unique peptides^*d*^	Total peptides^*e*^	AA^*f*^	Mol wt (kDa)
Q09666	AHNAK GN=AHNAK	6,349.70	72.41	209	302	5,890	628.7
Q8IVF2	AHNAK2 GN=AHNK2	1,610.09	36.15	50	81	5,795	616.2
P08195	CD98 GN=SLC3A2	309.22	23.81	9	13	630	68.0
P05556	Integrin β1 GN=ITGB1	123.99	8.52	5	5	798	88.4
O00401	NWASP GN=WASL	34.67	7.72	1	3	505	54.8
Q01650	LAT1 GN=SLC7A5	63.96	7.69	1	3	507	55.0
P16070	CD44 GN=CD44	73.46	4.99	2	3	742	81.5
O00592	Podocalyxin GN=PODXL	41.67	4.66	2	2	558	58.6
Q15758	ASCT2 GN=SLC1A5	60.98	4.44	2	2	541	56.6
Q96RU3	Formin BP1 GN=FNBP1	39.77	1.62	1	1	617	71.3
P29317	EphA2 GN=EPHA2	40.11	1.02	1	1	976	108.2
P00533	EGFR GN=EGFR	46.38	0.83	1	1	1,210	134.2

a Uniprot gene names are provided as a common designation followed by acronym.

b Confidence score expressed as cumulative mass spectra for detected peptides.

c Percentage of respective protein represented by cumulative detected peptides.

d Values correspond to the number of high-confidence peptides detected.

e Numbers correspond to the total number of peptides detected.

f Numbers correspond to the total number of amino acids in the respective proteins.

10.1128/mBio.02861-20.1 TABLE S1 Full listing of host proteins identified by Mascot data analysis of mass spectroscopy results. Proteins are listed in order of confidence score from replicate tests for proteins detected in the presence of BirA alone (sheets 1 and 2), TmeA-BirA (sheets 3 and 4), or mldTmeA-BirA (sheets 5 and 6). Identifying Uniprot accession numbers and protein descriptions are provided along with confidence assessment. The confidence score is expressed as cumulative mass spectra for detected peptides. The percentage coverage of respective proteins is indicated along with the number of unique and total peptides detected. Download Table S1, XLSX file, 0.07 MB. Copyright © 2021 Keb et al. 2021 Keb et al. This content is distributed under the terms of the Creative Commons Attribution 4.0 International license.

Reproducibility of TmeA-BirA-mediated biotinylation of these proteins was assessed via immunoblot analysis of biotinylated material derived from multiple experiments. A TmeB-BirA treatment was included as an additional negative control. These analyses (Fig. S3A) confirmed reproducible TmeA-dependent biotinylation of receptors CD44, EphA2, and EGFR but not ITGβ1, solute transporters SLC3A2, SLC7A5, and SLC1A5, and the actin-associated proteins AHANK and N-WASP. Consistent with their absence in MS data, AnnexinA2 and PDGFR were not detected. N-WASP has been implicated in association with sorting nexin 9 (SNX9) during chlamydial invasion (19), and SNX9 was also selectively biotinylated in the presence of TmeA. Gene ontology (Table 2) and STRING (Fig. S3B) (http://www.string-db.org; 41) analyses indicated several functional links among the targeted host proteins. Specific host proteins were clustered in functional classes related to amino acid transport and processes requiring manipulation of the host cytoskeleton. The detected amino acid and membrane-associated transport proteins SLC1A5, SLC7A5, and SLC3A2 appeared in multiple ontology categories. Overall, these functional classes point to an impact on the host actin network.

**TABLE 2:** Selected enriched gene ontology (GO) molecular functions identified among host proteins targeted by TmeA-BirA

Term	*P* value^*a*^	Specific targets
Amino acid transport	2.88e-05	SLC7A5, SLC3A2, SLC1A5
Organic acid transmembrane transport	2.69e-05	SLC7A5, SLC3A2, SLC1A5
Viral entry into host	1.11e-05	EphA2, EGFR, SLC1A5
Cell migration	4.28e-05	EphA2, SLC7A5, EGFR, CD44, SLC3A2
Movement of cell or subcellular component	2.97e-05	EphA2, SLC7A5, N-WASP, EGFR, CD44, SLC3A2

a Calculated *P* value using Fisher’s exact test and setting the false-discovery rate at <0.05.

10.1128/mBio.02861-20.4 FIG S3 Detection of biotinylated host proteins via immunoblot. (A) HeLa cells were mock-treated or nucleofected with Tme-BirA, ΔmldTmeA-BirA, or TmeB-BirA and cultivated for 24 h in the presence of biotin. Whole-culture material was harvested, biotinylated proteins were isolated with Avidin beads, and eluates were subjected to immunoblot analysis. HeLa lysates were also loaded as a control for antibody specificity. Representative images from a single experiment are shown. BirA-containing fusion proteins were detected with cMyc-specific antibodies. Host proteins were detected using antibodies indicated in Materials and Methods. (B) Reproducibly detected host proteins were examined via STRING analysis and grouped according to ontology analysis. Yellow corresponds to transmembrane transport, whereas the orange sphere reflects proteins involved in cell movement. Download FIG S3, TIF file, 0.4 MB. Copyright © 2021 Keb et al. 2021 Keb et al. This content is distributed under the terms of the Creative Commons Attribution 4.0 International license.

**FIG 2:**
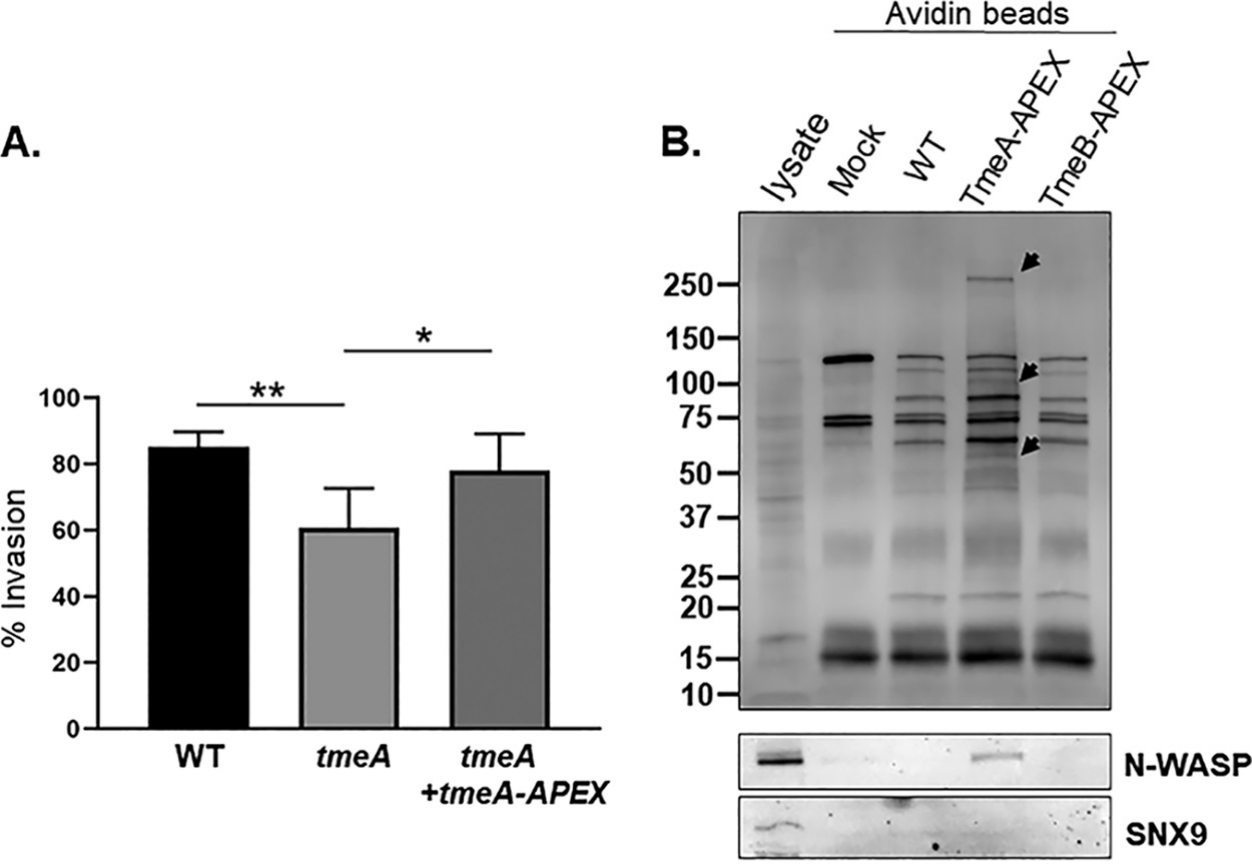
A TmeA-APEX fusion is functional when expressed in C. trachomatis and is able to biotinylate N-WASP. (A) The ability of TmeA-APEX to complement Δ*tmeA* invasion was tested by infecting HeLa monolayers for 1 h at 4°C with WT, *tmeA*, or *tmeA* expressing TmeA-APEX at an MOI of 10. Cultures were shifted to 37°C for 45 min and then paraformaldehyde fixed and processed for inside-out staining to assess invasion efficiency. Data are represented as percentage of internalized chlamydiae with standard deviations. Statistical significance was computed using Student’s *t* test with Welch’s correction (*, *P* < 0.003; **, *P* < 0.0001). (B) HeLa monolayers were mock treated or infected for 24 h with WT and Δ*tmeA* or Δ*tmeB* strains expressing TmeA-APEX or TmeB-APEX, respectively. Whole-culture material was harvested, biotinylated proteins were isolated with Avidin beads, and eluates were subjected to immunoblot analysis. HeLa lysates were also loaded as a control for antibody specificity. Total biotin content was probed using HRP-conjugated avidin; arrows mark the positions of proteins uniquely present in TmeA-APEX samples. N-WASP or SNX9 were detected using specific antibodies.

We utilized the engineered ascorbate peroxidase, APEX2, which biotinylates proximal proteins in the presence of biotin-phenol and hydrogen peroxide, to test whether the BirA-identified protein candidates were in proximity to TmeA in the context of chlamydial infection. In contrast to BirA, APEX2 is readily secreted through the T3SS (42). We generated expression plasmids for both TmeA-APEX and TmeB-APEX chimeric proteins and introduced them into the respective null C. trachomatis strains. We utilized the well-established invasion defect of Δ*tmeA* (34) to determine whether TmeA-APEX is functional. HeLa cells were synchronously infected with either the WT, Δ*tmeA*, or Δ*tmeA+tmeA-APEX*, and the percentage invasion was quantified for each strain using differential inside-out fluorescence staining 45 min postinfection. No significant difference in invasion efficiency was detected between WT and *tmeA+tmeA-APEX* strains (Fig. 2A), indicating functionality of the TmeA-APEX chimera. In control experiments, we were unable to generate sufficient biotinylation signal at infection time points corresponding to invasion and entry (data not shown); therefore, the profile of biotinylated proteins was examined 24 h postinfection. HeLa cells were infected with mock, WT, or APEX strains and subsequently cultivated for 30 min with biotin-phenol-supplemented medium and then treated with hydrogen peroxide to catalyze the biotinylation of proximal proteins. Whole-culture material was collected, biotinylated proteins were affinity precipitated with avidin resin, and recovered proteins were analyzed via immunoblot. Consistent with functional APEX activity, we detected several uniquely biotinylated proteins in the presence of TmeA-APEX in addition to naturally biotinylated host and/or chlamydial proteins (Fig. 2B). Two of the uniquely biotinylated bands were in the size range of N-WASP and SNX9 (ca. 65 kDa and 90 kDa, respectively) (13, 19). Immunoblots with protein-specific antibodies revealed the unique presence of N-WASP, but not SNX9, in TmeA-APEX material.

**FIG 3:**
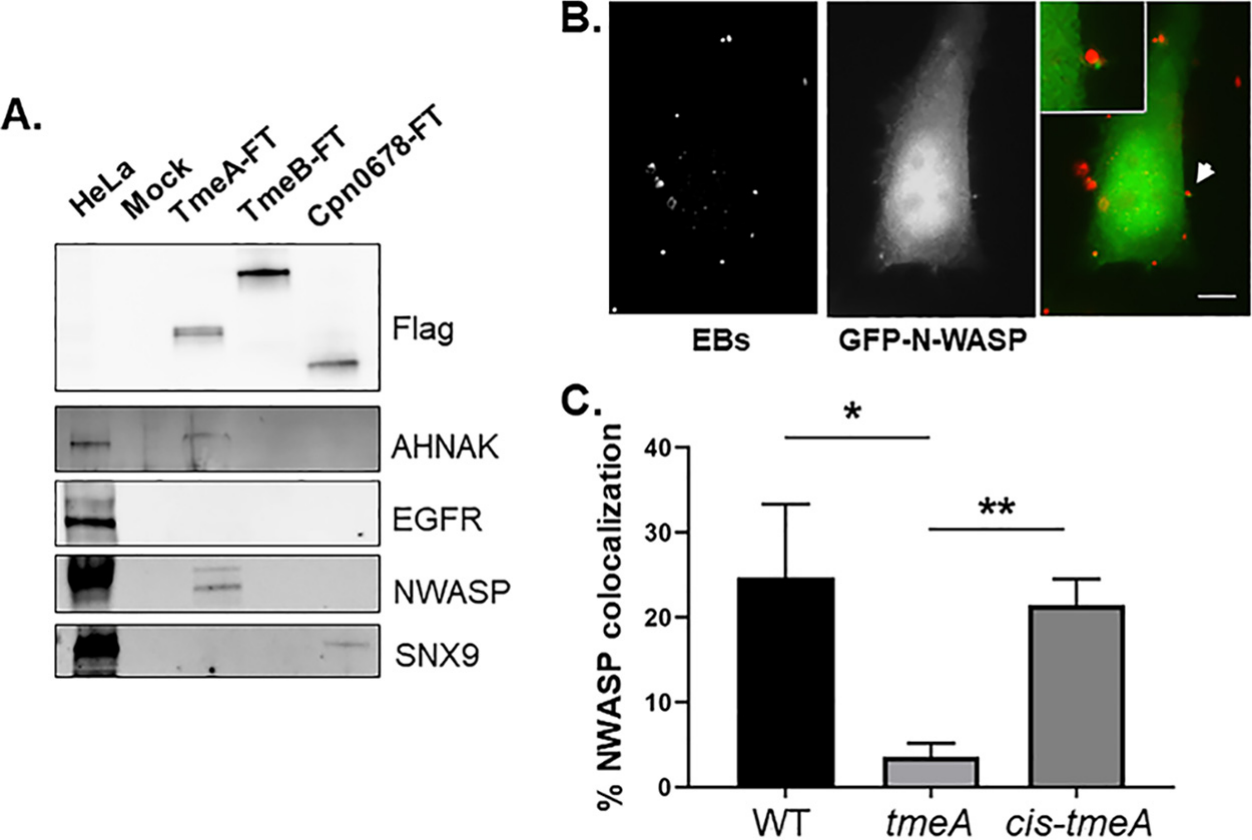
TmeA interacts with N-WASP, and N-WASP recruitment to sites of invading EBs requires TmeA. (A) Flag-tagged proteins were immunoprecipitated from whole-cell lysates of HeLa cells expressing TmeA-FT, TmeB-FT, or Cpn0678-FT for 24 h. Immunoprecipitation from mock-treated lysates served as a negative control. Eluted material was probed in immunoblots for tagged chlamydial proteins using anti-Flag antibodies. Host proteins were detected using antigen-specific antibodies, and HeLa whole-cell lysates were included as a positive control for these antibodies. (B). GFP-N-WASP (green)-expressing HeLa cells were infected for 10 min with WT C. trachomatis (red) and visualized by epifluorescence microscopy. The arrow indicates the field of view shown as an inset in the merged image. Bar = 5 µm. (C). HeLa cells were cultivated for 20 min after synchronous infection (in triplicate) with WT, Δ*tmeA*, or cis-*tmeA* strains. Monolayers were stained for NWASP and *Chlamydia* using specific antibodies, and the percentage of colocalization was enumerated for ca. 100 randomly selected EBs. Data are represented as the percentage of EBs exhibiting adjacent NWASP localization. Statistical significance was computed using Student’s *t* test with Welch’s correction (*, *P* < 0.04; **, *P* < 0.002).

Both BirA and APEX2 proximity labeling approaches identified N-WASP as proximal to TmeA. The possibility that TmeA directly interacts with N-WASP was tested by ectopically expressing flag-tagged (FT) TmeA in HeLa cells and performing coimmunoprecipitation experiments. We also examined SNX9 as a control. 24 h postnucleofection, whole-cell material was collected, and FT proteins were immunoprecipitated. Immunoblots were used to detect host proteins precipitated with TmeA-FT or TmeB-FT (Fig. 3A). C. pneumoniae Cpn0678 was used as a positive control for SNX9 interaction. N-WASP coprecipitated with TmeA-FT, while SNX9 did not. N-WASP also significantly colocalized with EBs in a TmeA-dependent manner during invasion of HeLa cells (Fig. 3B). HeLa cells were nucleofected to ectopically express N-WASP-green fluorescent protein (GFP) and then infected 24 h post nucleofection with either the WT, Δ*tmeA*, or Δ*tmeA+cis-tmeA.* Then, 10 min postinfection, cultures were fixed, and EBs were stained for detection by fluorescence microscopy (Fig. 3B). Colocalization was also apparent using N-WASP-specific antibodies, and indirect immunofluorescence indicated a peak colocalization of TmeA and N-WASP at 20 to 30 min postinfection (data not shown). The dependence of TmeA on N-WASP recruitment was tested by enumerating the number of EBs associated with N-WASP comparing WT and Δ*tmeA* strains. A cis-complemented *tmeA* strain expresses WT levels of TmeA and restores invasion efficiency comparable to that of the WT (Fig. S4). When HeLa cells were infected for 20 min, N-WASP colocalized with ca. 20% of WT and cis-*tmeA* EBs, whereas colocalization was <4% for Δ*tmeA* (Fig. 3C). These data indicate that TmeA interacts with and recruits N-WASP during invasion.

10.1128/mBio.02861-20.5 FIG S4 *Cis*-complementation of Δ*tmeA* restores WT levels of TmeA and invasion efficiency. (A) Protein from equivalent IFU of DG-purified WT, ΔtmeA, and cis-*tmeA* EBs was concentrated and resolved for immunoblotting to compare relative levels of TmeA. TmeA was detected via specific antibodies, and detection of C. trachomatis Hsp60 was used as a loading control. (B) The ability of *cis*-*tmeA* to complement the Δ*tmeA* invasion phenotype was tested by infecting HeLa monolayers for 1 h at 4°C with the WT, *tmeA*, or cis-*tmeA* at an MOI of 10. Cultures were paraformaldehyde fixed and processed for inside-out staining to assess invasion efficiency after 45 min of incubation at 37°C. Data are represented as the percentage of internalized chlamydiae with standard deviations. Statistical significance was computed using Student’s *t* test with Welch’s correction (*, *P* < 0.003; **, *P* < 0.0001). Download FIG S4, TIF file, 0.1 MB. Copyright © 2021 Keb et al. 2021 Keb et al. This content is distributed under the terms of the Creative Commons Attribution 4.0 International license.

**FIG 4:**
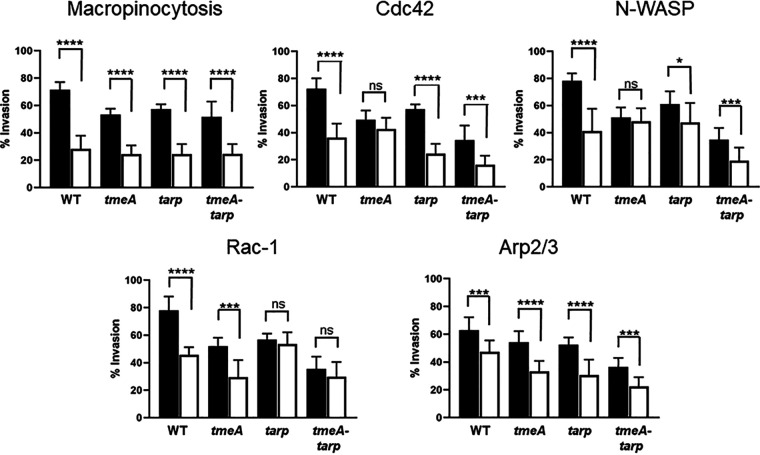
TmeA function is associated with Cdc42 and N-WASP activity, whereas TarP is differentially associated with Rac1. HeLa monolayers were infected for 1 h at 4°C with WT or mutant strains at an MOI of 10. Infections were carried in the absence (black bars) or presence (white bars) of specific inhibitors. Pharmacologic disruption of macropinocytosis, Cdc42, N-WASP, Rac1, or Arp2/3 was achieved using 100 µM EIPA, 20 µM casin, 25 µM wiskostatin, 25 µM Ehop-016, or 200 µM CK666, respectively. Cultures were shifted to 37°C and maintained for 45 min, with or without drug, and then paraformaldehyde fixed and processed for inside-out staining to assess invasion efficiency. Data are represented as mean values for the percentage of internalized chlamydiae and are shown with standard deviations. Statistical significance was computed using Student’s *t* test with Welch’s correction (*, *P* < 0.002; ***, *P* < 0.0004; ****, *P* < 0.0001).

The invasion defect manifested by Δ*tmeA-tarp* is consistent with synergistic roles of TmeA and TarP during chlamydial entry, and we next wanted to examine the potential pathways involved. Pharmacologic inhibitors that disrupt relevant pathways relating to cytoskeletal rearrangements were used to gain insight into the mechanistic contributions of TmeA and TarP (Fig. 4). The percentage invasion was determined by differential inside-out staining for both treated and untreated monolayers comparing WT and mutant strains. Host cells were treated for 15 min prior to infection, infected by rocking on ice for 1 h, and then shifted to 37°C for 45 min before fixation with paraformaldehyde. EIPA (5-[*N*-ethyl-*N*-isopropyl] amiloride), a Na^+^/H^+^ exchange inhibitor, was used to block macropinocytosis but not receptor-mediated endocytosis (43). All strains were susceptible to EIPA treatment and had significantly decreased invasion efficiency. Cdc42 and Rac1 were specifically targeted with casin and EHop-016, respectively. Interestingly, Δ*tmeA* invasion was not significantly affected in response to Cdc42 inhibition, nor was it susceptible to N-WASP inhibition (Fig. 4). In contrast, Δ*tarp* invasion was not impacted by Rac1 inhibition during invasion. All strains were susceptible to Arp2/3 inhibition by CK666. Where appropriate, cis-complemented strains were used to infect drug-treated or untreated monolayers to confirm that the lack of inhibitor susceptibility was due to a loss of TmeA or TarP (Fig. S5). In all cases, *cis*-complementation restored susceptibility to the respective drugs. These data suggest that TmeA and TarP are uniquely involved in Cdc42/N-WASP and Rac1 pathways, respectively, but their functions likely converge with downstream activation of Arp2/3. Interestingly, susceptibility of the *tmeA-tarp* mutant strain to Rac1, Cdc42, and N-WASP inhibition all mirrored the phenotype of the Δ*tarp* strain. These results are consistent with exerting a dominant, upstream function during invasion.

10.1128/mBio.02861-20.6 FIG S5 Complementation of mutants restores invasion efficiency and WT sensitivity to the respective inhibitors. Invasion assays were carried out in the absence (black bars) or presence (white bars) of drugs, and levels of invasion for the WT were compared to the respective null mutants *cis*-complemented with full-length *tarp* or *tmeA*. Assays were carried out with 25 µM Ehop-016 (Rac1) for *cis*-*tarp* and 20 µM casin (Cdc42) or 25 µM wiskostatin (N-WASP) for *cis*-*tmeA*. Data are represented as mean values for the percentage of internalized chlamydiae and are shown with standard deviations. Statistical significance was computed using Student’s *t* test with Welch’s correction (***, *P* < 0.0004; ****, *P* < 0.0001). Download FIG S5, TIF file, 0.09 MB. Copyright © 2021 Keb et al. 2021 Keb et al. This content is distributed under the terms of the Creative Commons Attribution 4.0 International license.

**FIG 5:**
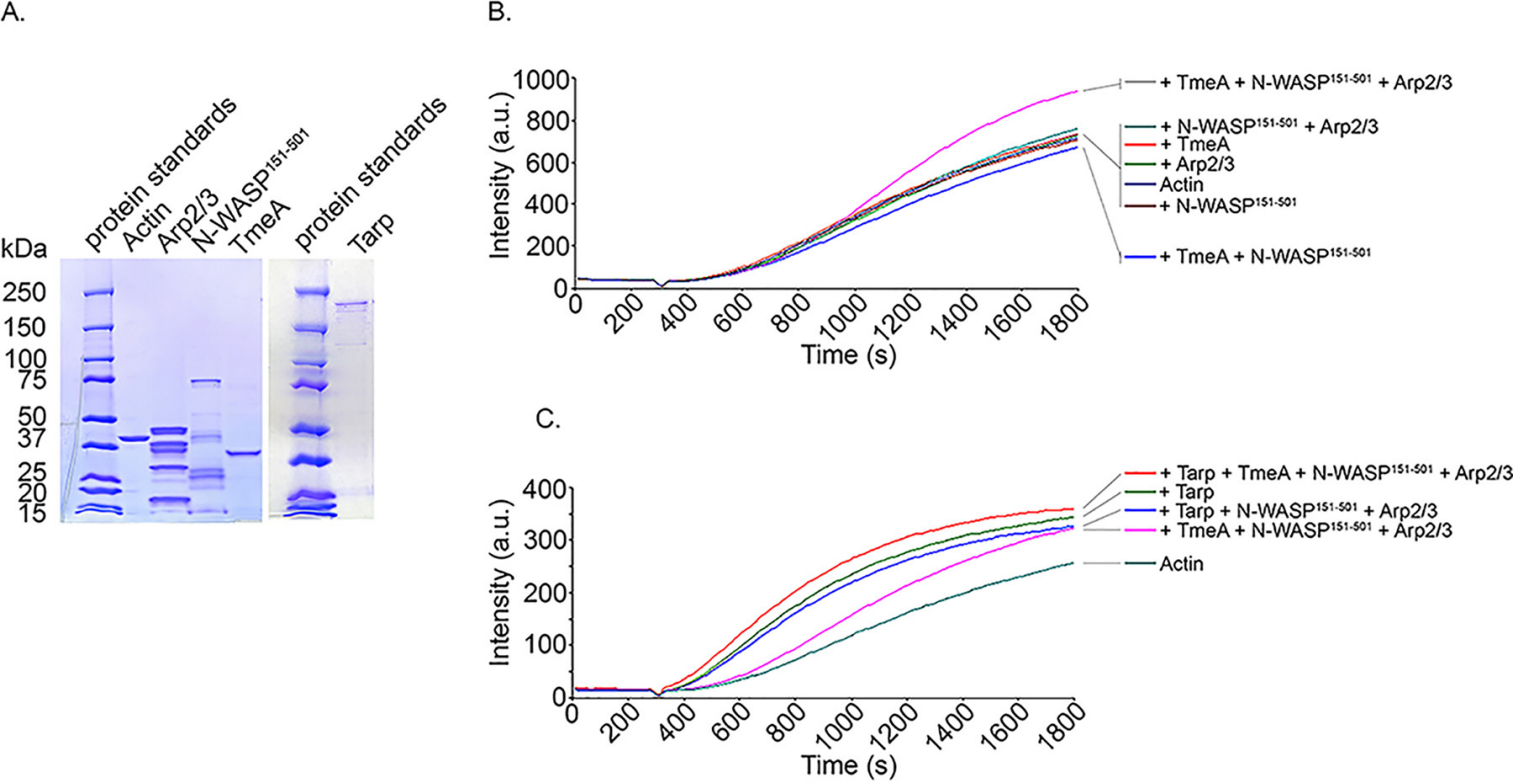
TmeA activates NWASP-Arp2/3-dependent actin polymerization, and rates are further enhanced in the presence of TarP. (A). Actin, TmeA, TarP, Arp2/3, and N-WASP^151-501^ proteins employed in the pyrene actin polymerization assay were resolved by SDS-PAGE and visualized by Coomassie blue staining. (B). TmeA, Arp2/3, and N-WASP were added individually or in combination to monomeric pyrene-labeled actin. A TmeA-mediated increase in actin polymerization after the addition of polymerization buffer at 300 s was measured as the arbitrary fluorescence intensity (arbitrary units [a.u.] over time [s]) with excitation and emission wavelengths of 365 and 407 nm, respectively. (C). Like the assay shown in panel B, with the addition of the actin nucleating effector TarP. Enhanced pyrene actin polymerization was measured in the presence of TarP and TmeA-N-WASP-Arp2/3.

Finally, we investigated whether the interaction with TmeA manifests as activation of N-WASP activity to promote actin polymerization through Arp2/3. An established *in vitro* assay leveraging pyrene-conjugated actin was employed to examine the kinetics of actin polymerization (44) in the presence of selected proteins. In physiological buffers, G actin spontaneously assembles into filaments but is limited by instability of actin dimers and trimers, thus preventing rapid elongation (45, 46). Actin nucleators, such as TarP, display a shortened or nonexistent lag phase. GST-tagged proteins were purified, and the tag was cleaved from TmeA to prevent the possibility of GST dimerization. Analysis of proteins in Coomassie-stained material indicated homogeneous content for the respective proteins (Fig. 5A). In Pyrenes assays (Fig. 5B), neither TmeA alone or in combination with N-WASP, nor N-WASP and Arp2/3 without TmeA, resulted in polymerization kinetics differing from actin alone. The rate of actin polymerization was clearly enhanced in the presence of TmeA, N-WASP, and Arp2/3, indicating that TmeA interaction with N-WASP activates N-WASP activity and thus leads to Arp2/3 activation and increased actin polymerization kinetics (Fig. 5B). We next tested whether actin polymerization kinetics could be further enhanced by the addition of TarP. Indeed, the combination of TarP, TmeA, N-WASP, and Arp2/3 resulted in more rapid actin polymerization kinetics (Fig. 5C); therefore, TarP and TmeA are capable of acting synergistically to polymerize actin.

## DISCUSSION

**FIG 6:**
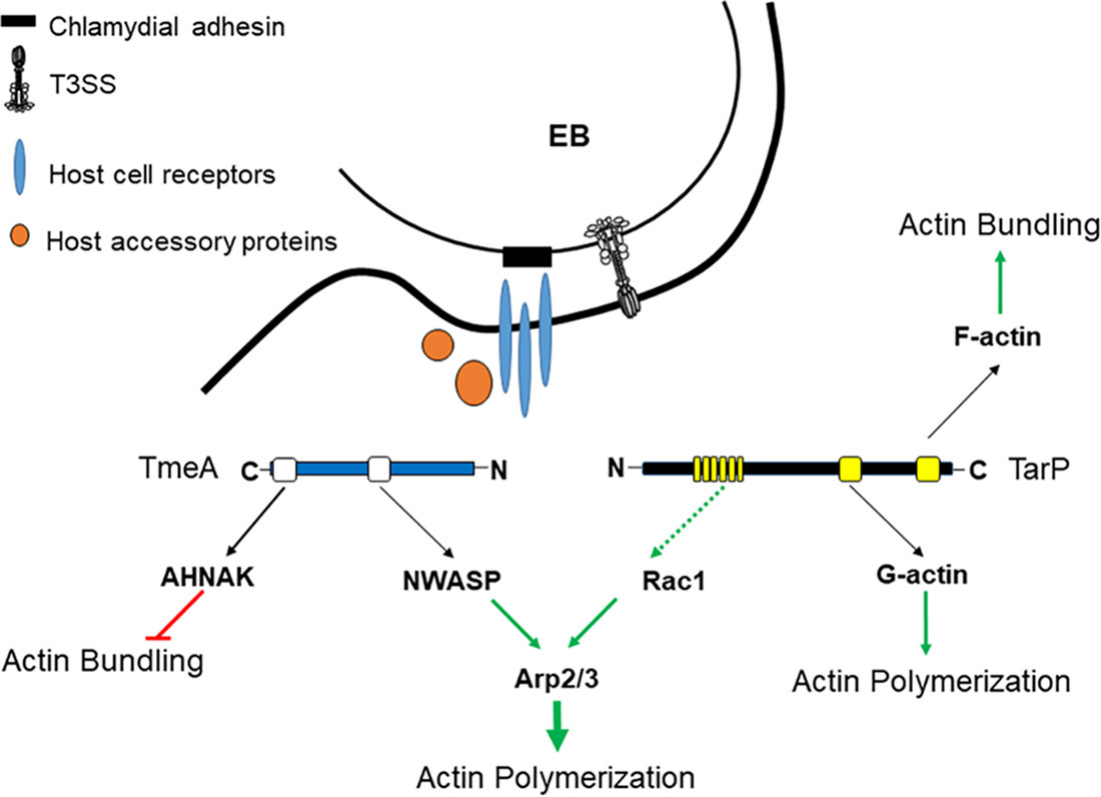
Schematic of a working model describing TmeA and TarP actin-dependent contributions to chlamydial invasion. Both TmeA and TarP are deployed to host cells via the T3SS after irreversible binding of EBs through adhesion (black) interactions with host cell receptors (blue) and recruitment of accessory host proteins (orange). Multiple actin-impacting domains are present in both TmeA (white boxes) and TarP (yellow boxes). TmeA negatively impacts actin bundling through a direct interaction with host cell AHNAK and promotes (green arrow) Arp2/3-dependent actin polymerization by activating N-WASP. TarP directly binds actin to promote polymerization and bundling through discrete domains that associate with G-actin or F-actin, respectively. TarP can also induce actin polymerization indirectly (dashed green line) via recruitment of factors leading to activation of Rac1 and subsequent Arp2/3-dependent actin polymerization.

The T3S chaperone Slc1 directs secretion of at least four effectors during chlamydial entry into epithelial cells, including TarP, TepP, TmeA, and TmeB (29). It is well established that C. trachomatis TarP influences actin polymerization both directly via actin nucleation/polymerization/bundling and in concert with host factors Rac1 and Arp2/3 (reviewed in reference 11). Although TmeA is also required for chlamydial entry and impacts actin dynamics, the molecular mechanisms manifesting the invasion function are less clear. TmeA associates with the host plasma membrane via the MLD (residues 40 to 80), which is functionally interchangeable with the MLD domains of *Yersinia* and *Pseudomonas* effectors YopE and ExoS, respectively (32). The C terminus of TmeA interacts with host AHNAK, and both TmeA and AHNAK localize adjacent to invading EBs (32, 34). Although TmeA interferes with the F-actin bundling activity of AHNAK, this activity is not responsible for the observed invasion defect manifested by the Δ*tmeA* strain (34). A TargeTron gene disruption of *tepp* reportedly did not impact invasion, yet data were not shown (47). We present evidence here that supports a working model whereby TmeA associates with the infection synapse formed between the host cell and an invading EB and initiates Arp2/3-mediated actin polymerization independently of TarP (Fig. 6). This model places TmeA downstream of chlamydial attachment and indicates that TmeA is responsible for direct activation of N-WASP to promote entry. Our data (Fig. S1C) also formally exclude the possibility that TepP and TmeB are essential for the invasion process. Simultaneous to preparation of our work, Faris et al. (48) reported an N-WASP binding domain within TmeA and implicated TmeA-dependent N-WASP activation of Arp2/3 during invasion of host cells by C. trachomatis. Our data are in general agreement with, but significantly extend, those observations.

Chlamydial invasion requires irreversible attachment of EBs followed by cytoskeletal rearrangements to trigger entry. Cumulative evidence indicates that manipulation of actin can be orchestrated both by activation of cell surface receptors and directly via the action of secreted effector proteins (11). Interestingly, ectopically expressed TmeA-BirA reproducibly resulted in biotinylation of CD44, EGFR, and EphA2 cell surface receptors. While the hyaluronic acid receptor CD44 has not been associated with chlamydial infectivity, host cell receptor tyrosine kinases EGFR (18, 49) and EphA2 (6) have been implicated for C. trachomatis. These receptors are activated in response to C. trachomatis infection via tyrosine phosphorylation and are separately important for chlamydial attachment and invasion. EphA2 (6) becomes activated within minutes of infection, whereas EGFR (18) activation is not apparent until after ca. 2 h. Both receptors later associate with the chlamydial inclusion, where they are essential for development, and inclusion-localized EGFR colocalized with F-actin assembly (18). Disruption of receptor tyrosine kinase activation is not expected, since loss of TmeA does not interfere with inclusion development (34). Indeed, loss of TmeA did not alter tyrosine phosphorylation or abundance of these receptors during entry or at later time points (data not shown). This would be in agreement with observations that EphA2 is upregulated during infection by the ERK pathway (6).

We did not detect PDGFRβ or reproducibly detect ITGβ1 by proximity labeling, both of which have been shown to promote C. trachomatis attachment and entry (15, 17), yet detection of amino acid transport proteins that are relevant to chlamydial infection was apparent. SLC3A2 (CD98hc) and SLC7A5 (LAT1) form the heterodimeric glycoprotein CD98, which is capable of regulating ITGβ1 in epithelial cells (50). The C. trachomatis adhesion Ctad1 engages ITGβ1 to promote attachment and entry (15), raising the possibility that CD98 is relevant to *Chlamydia* infection. The glutamine transporter SLC1A5 (ASCT2) has not been implicated during entry but is essential for glutamine-dependent survival of intracellular chlamydiae (51). We acknowledge that proximity to receptor and SLC proteins occurred in the absence of *Chlamydia* infection but note that host protein labeling required localization to the plasma membrane since they were not biotinylated in the presence of MLD-deficient TmeA-BirA. These data are in contrast to labeling of AHNAK, which did not require the MLD, and raise the possibility that these TmeA associations have spatial requirements. The potential relevance and role of TmeA localizing near these proteins clearly requires further study. In aggregate, we speculate that TmeA functions downstream of cell surface receptors and does not impact their activity.

Labeling of N-WASP also required the TmeA MLD in BirA proximity labeling studies. These data led us to test for physical interactions of TmeA with N-WASP using coimmunoprecipitation. Indeed, N-WASP coprecipitated specifically with Flag-tagged TmeA expressed in HeLa cells. Whether this interaction was manifested during infection was also examined. We agree with Faris et al. (48) that the previously observed recruitment of N-WASP to EBs (19) requires TmeA and is transient during the entry process. In our hands, colocalization of N-WASP with WT EBs was below detection by 30 min postinfection (data not shown). Our data revealed EB-adjacent foci of N-WASP that were most evident when bacteria associated with edges and surface projections that would be consistent with the proposed filopodial capture of *Chlamydia* (19). Proximity labeling using a TmeA-APEX2 fusion expressed in *Chlamydia* was also used to confirm association of TmeA and N-WASP in the context of infection. Although the fusion protein complemented the *tmeA* invasion defect, abundance and sensitivity issues confounded our efforts to capture potential TmeA-N-WASP proximity during the 15- to 20-min window of the invasion process. Experiments were therefore performed at 24 h postinfection when TmeA-APEX2 would be present in abundance. Under these conditions, N-WASP was detected in biotin-labeled fractions. Interestingly, these APEX2 data suggest that TmeA can maintain or reestablish an interaction with N-WASP during later stages of infection. Developing inclusions are enveloped in dynamic actin cages that act as scaffolds and confer stability (12, 52). Neither the N-WASP nor downstream actin branching protein complex Arp2/3 are required for this actin coat assembly (52); yet N-WASP and actin polymerization are required later for subsequent host cell exit via the extrusion mechanism (13). Extrusion is a complex process involving both host factors and chlamydial T3S effectors (reviewed in reference 11). Importantly, TmeA is also secreted during late-cycle development (35) where the MLD would target this pool of TmeA to the plasma membrane. Indeed, a split GFP technique revealed accumulation of TmeA at the plasma membrane of infected cells harboring mature inclusions (53). It is therefore tempting to speculate that this pool of TmeA may contribute to extrusion via activation of N-WASP.

Identifying N-WASP as an additional target of TmeA led us to more closely investigate macropinocytosis, a newly appreciated mechanism for internalization of chlamydiae that requires N-WASP activity (19). We chose to examine pharmacologic inhibitors previously implicated in macropinocytotic internalization of C. trachomatis (19), and our comparative sensitivity data reinforced working models and provided interesting new insights. Invasion of single and double *tmeA* and *tarp* mutants were susceptible to EIPA comparable to the WT. This observation is consistent with our model since EIPA inhibits macropinocytosis by lowering submembranous pH, preventing signal transduction through both Cdc42 and Rac1 (54). Mutant strain invasion efficiency was also similarly reduced compared to that of the WT in the presence of CK666 and supports the proposed model (48) where TarP and TmeA’s functions converge at Arp2/3. Strains lacking *tarp* or *tmeA* lost sensitivity to inhibition with Ehop-016 and wiskostatin, respectively. Hence, Rac1 function is important for TarP-mediated invasion, whereas TmeA-mediated invasion functions through N-WASP. All strains, except those which lacked only *tmeA*, were susceptible to the Cdc42 inhibitor casin. A role for Cdc42 in C. trachomatis entry was originally ruled out based on a lack of robust colocalization of Cdc42 with invading EBs, the absence of Cdc42 detection using coprecipitation with the CRIB domain of Pak1, and insensitivity of chlamydial entry to overexpression of dominant negative Cdc42 (55). However, Ford et al. (19) noted early colocalization of GFP-Cdc42 with invading EBs and a modest sensitivity of invasion to casin, raising the possibility that Cdc42 has a transient role. Our data are consistent with the latter case. The robust level and extended duration of Rac1 activation (55) could indicate a comparatively more extensive role of Rac1 in chlamydial infection. The *tmeA* mutant strain also lost susceptibility to casin. We did not examine Cdc42 localization or activity here because the pyrene assay data indicate TmeA bypasses the need for Cdc42 in N-WASP activation. Finally, we noted that drug sensitivity of the double mutant always mirrored that of the *tarp* mutant strain. These data may indicate a dominant role for Tarp during entry. However, *tmeA* and *tarp* mutant strains are equally deficient for entry in the absence of inhibitor; thus, additional work is required to delineate the comparative roles of these effectors.

Faris et al. (48) identified a specific domain of TmeA (residues 118 to 126), resembling the GBD ligand motif found in the enterohemorrhagic Escherichia coli effector EspFu, responsible for interacting with the GBD domain of N-WASP. They surmised that this interaction leads to activation of N-WASP and subsequent Arp2/3-dependent actin polymerization. The EspFu GBD ligand motif associates with the N-WASP GBD domain similarly to Cdc42 (56). EspFu has therefore been proposed to mimic the N-WASP-activating activity of Cdc42 by inducing conformational changes necessary for N-WASP activation of Arp2/3-dependent actin polymerization (56). We provide direct evidence here that TmeA is sufficient to activate N-WASP, raising the possibility that TmeA also acts as a Cdc42 mimic. Indeed, *in vitro* reaction mixtures containing TmeA in combination with N-WASP and Arp2/3 resulted in elevated rates of pyrene fluorescence indicative of actin polymerization. This polymerization was synergistic with TarP’s endogenous actin polymerization activity. Therefore, TmeA’s interaction with N-WASP is sufficient to activate association with Arp2/3 and contributes in an additive fashion with TarP to the actin polymerization necessary to promote C. trachomatis invasion.

Finally, SNX9 is another essential component of macropinocytosis-mediated entry and has been implicated for invasion of both C. trachomatis (19) and C. pneumoniae (22). Although SNX9 was detected in our BirA proximity labeling experiments via Western blotting, we did not detect evidence of an interaction of TmeA with SNX9. C. pneumoniae Cpn0678 and C. trachomatis TmeA lack homology but are encoded in the same genomic locus positioned immediately upstream of *tmeB* (33). Cpn0678 binds directly to SNX9 (22). In our studies, Flag-tagged Cpn0678, but not TmeA, coprecipitated with SNX9. This is consistent with primary sequence analysis indicating that TmeA lacks the apparent proline-rich motifs found in Cpn0678 that mediate the interaction with SNX9. The proline content of TmeA is 4.3% with residues spaced throughout the protein, whereas Cpn0678 contains 12.2% proline with 3 proline-rich repeats spanning residues 137 to 213. Cpn0678 did not appear to interact with N-WASP or AHNAK, and these data emphasize an instance where chlamydial species are functionally divergent. SNX9 was associated with host membrane curvature in the case of C. pneumoniae (22) and filopodial capture (19) during C. trachomatis infection. Based on electron microscopy data, Faris et al. (48) indicated a requirement of TmeA in filopodia formation. This observation would be consistent with robust induction of surface structures induced by high-multiplicity of infection (MOI) chlamydial infection (9), but it is unclear how this fits with data indicating that *Chlamydia* hijacks existing macropinocytosis filopodia instead of inducing the *de novo* assembly of the structures (57). Perhaps functionally distinct protrusions are being manifested when *Chlamydia* associates with host cells.

It is clear that TmeA and TarP represent two chlamydial effectors that have an intimate and complex relationship. Efficient TarP-mediated entry requires the C-terminal filamentous-actin binding domain most prominently and the tyrosine-containing repeat domain to a lesser extent (28). The overt role of TmeA during invasion involves activation of N-WASP to promote Arp2/3-dependent actin polymerization. Overall, our data support the notion that TmeA and TarP have distinct functions yet synergistically promote chlamydial invasion by facilitating actin polymerization associated with the macropinocytosis pathway. Moreover, we have demonstrated how markerless gene deletion via FLAEM can be leveraged to generate multimutant strains. Our previous work has revealed situations where nonphysiological levels of expression typical with *trans*-complementation schemes can complicate data (34). *Cis*-complementation overcomes this confounding challenge, and we further demonstrate the efficacy of *cis*-complementation using allelic replacement in this study. Our work, therefore, also establishes how evolving and improving genetic approaches now facilitate detailed molecular dissection of effector function in *Chlamydia.*

## MATERIALS AND METHODS

### Cell culture and organisms.

C. trachomatis serovar L2 (LGV 434) and derivative strains were used in these studies. Previously described strains include C. trachomatis *tarp* (28), *tmeB* (36), and *tmeA-lx* (37) (referred to here simply as Δ*tmeA*). C. trachomatis *tepp* was generated via FRAEM (36) and will be described in detail elsewhere. CaCl_2_-mediated chemical transformation (58) was used to mobilize respective plasmids into C. trachomatis L2. Subsequent manipulations leveraging fluorescence reporting to yield *trans*-expression or allelic replacement were accomplished according to established protocols (28, 59, 60). Chlamydiae were routinely maintained in either HeLa 229 epithelial cell monolayers (CCL-1.2; ATCC) or McCoy cell monolayers (CRL-1696; ATCC). Unless otherwise indicated, all cultures were grown in RPMI 1640 medium containing 2 mM l-glutamine (Life Technologies) supplemented with 10% (vol/vol) heat-inactivated fetal bovine serum (FBS; Sigma) at 37°C in an environment with 5% CO_2_ and 95% humidified air. All infections were accomplished using density gradient-purified EBs (61) centrifuged onto cell monolayers at 20°C for 1 h at 900 × *g* or rocking on ice when appropriate. For transformation and FRAEM protocols, chlamydiae were cultivated in the presence of 600 ng/ml penicillin G (PenG; Sigma), 500 µg/ml spectinomycin (Spec; AlfaAesar), 1 µg/ml cycloheximide (Sigma), and 50 ng/ml anhydrotetracycline (ATc) where appropriate. Rifampin (Rif)-resistant strains were generated as described previously (62) by cultivation for 4 passages in 2.5 ng/ml Rif, followed by 4 passages in 5 ng/ml Rif. Clonal isolates for all final *Chlamydia* strains were obtained as described by 2 sequential limiting dilution passages in 384 plates (60). Primary infections were carried out using particle- or inclusion-forming unit (IFU)-normalized chlamydiae, as indicated.

### DNA methods.

BirA-containing expression constructs were generated by mobilizing full-length C. trachomatis L2 *tmeA* or *tmeB* and recombinant *tmeA*Δ*mld* (32) into pcDNA3.1 mycBioID (32, 63). Custom primers (Integrated DNA Technologies [IDT]) containing engineered flanking KpnI sites were used to amplify *tmeA* or *tmeB*. Amplification of *tmeA* sequences was accomplished using 5′ (CCGGTACCGAGTATTCGACCTACTAATGGGAGTGGAAATG) and 3′ (GGGGGTACCTTAGTCTAAGAAAACAGAAGAAGTTATGACAGTTAGTGTTTGG) primers, whereas *tmeB* was amplified with alternative 5′ (CCCGGTACCGAGTAGCATAAGCCCTATAGGGGGG) and 3′ (GGGGGTACCTTAGATATTCCCAACCGAAGAAGGATCTTCCTC) primers. Standard cloning procedures were employed to insert chlamydial genes into the KpnI site of pcDNA3.1 mycBioID to yield chimeric sequences encoding N-terminally tagged TmeA, TmeAΔmld, or TmeB.

APEX2-containing constructs were generated by first amplifying APEX2 from pcDNA3 APEX2-NES (64) using custom primers (IDT) 5′(GACTACAAGGATGACGACGATAAGGGAAAGTC) and 3′ (CCCTCTAGATGCATGCTCGAGCTATTAGTC) and mobilizing the fragment into pBOMB-4 (65) between the mCherry and aadA sequences. Next, full-length *tmeA* or *tmeB*, excluding the stop codon, was amplified from the C. trachomatis L2 genome and mobilized into pBomb-APEX immediately upstream of APEX and replacing mCherry via iPCR. TmeA was amplified using 5′ (GAAAGGATCTGCGGCCGCATGAGTATTCGACCTACTAATGGGAGTGGAAAT) and 3′ (CTTTCCCTTATCGTCATCCTTGTAGTCGTCTAAGAAAACAGAAGAAGTTATGACAGTTAGTGTTTGG) custom primers. TmeB was amplified using 5′ (GATCTGCGGCCGCATGAGTAGCATAAGCCCTATAGG) and 3′ (CTTTCCCTTATCGTCGTCATCCTTGTAGTCGATATTCCCAACCGAAGAAGGAT) custom primers.

The plasmid used to generate the *tmeA cis*-complemented strain was generated using a plasmid constructed via the Gibson assembly using HiFi DNA assembly master mix (New England Biolabs). A two-step process was employed where an ca. 5.5-kb fragment containing *tmeA*, *tmeB*, and ca. 2 kb upstream of *tmeA* was amplified from WT L2 C. trachomatis via PCR using primers 695Cis5armF2 (gtcaCTGCAGGTACCGGGACACTCTATCCCCAAAGTTATTCTTCAAAAGTTCT) and 695Cis5armR2 (aggcatgatgatGAATGGTCGATTAGATATTCCCAACCGAAGAAGGATCTTC) and was mobilized into the SalI site of pSUmC-aadA (59) such that the chlamydial DNA was positioned immediately upstream of the *aadA* promoter. Then, 3 kb of DNA downstream of *tmeB* was amplified from WT L2 C. trachomatis via PCR using primers 695 cis 3armF (CTCACTGATTAAGCATTGGTAACCTGGGTTCCGCGCACATTTCC) and 695 cis 3armR (CTTTCTACGGGGTCTGACCTTTGCTTGCTCCCAAATTGTAAACGC). A Gibson reaction was used to mobilize this element into the SbfI site immediately downstream of *aadA*.

### Immunodetection and microscopy.

For immunoblot analyses, proteins were separated on 4% to 15% SDS-PAGE gels (Bio-Rad) and transferred to 0.45-µm polyvinylidene difluoride (PVDF) membranes (Millipore). The primary antibodies used were MOMP (33); TmeA (33); TmeB (35); TarP (8); TepP (kindly provided by Raphael Valdivia, Duke University); AHNAK (33); Scc2 (66); Flag-Tag (Sigma); Myc-Tag (Rockland); EGFR (Santa Cruz Biotechnology); chlamydial lipopolysaccharide (LPS) (NOVUS); CD44, EphA2, tubulin, ITGB1, WASL/N-WASP, SLC7A5, SLC1A5, and PDGFR (Cell Signaling); SLC3A2, PODXL, FNBP1, and SNX9 (Invitrogen); and phospho-tyrosine (4G10, Millipore). We used peroxidase-conjugated secondary antibodies (Sigma) and Amersham ECL Plus (GE Healthcare UK Limited) detection reagent. Biotinylated proteins were detected using Avidin horseradish peroxidase conjugate (Invitrogen). Fluorescence detection via microscopy was accomplished using direct fluorescence of HeLa cells expressing GFP-N-WASP (67) or by indirect immunofluorescence using primary antibodies specific to MOMP (Novus Biologicals) or N-WASP (Cell Signaling) and secondary antibodies conjugated to Alexa Fluor-594 or -488 (Invitrogen). Cells were examined via epifluorescence microscopy, and where appropriate, images were acquired using a ×100 oil-immersion objective. Images were processed equivalently using Adobe Photoshop 6.0 (Adobe Systems).

### Subcellular fractionation.

Separation of soluble and membrane-associated proteins was performed as described (32). Deoxyguanosine (DG) purified EBs or HeLa monolayers synchronously infected for 30 min with equal IFUs of WT or mutant strains were lysed in 1.5 ml of ice-cold 1% Triton lysis buffer (1% Triton X-114 [Sigma], 100 mm KCl, 50 mm Tris-HCl [pH 7.4]), rotated for 30 min at 4°C, and clarified by centrifugation. Aqueous and detergent phases were separated via differential temperature treatments and subjected to back extraction four times to yield homogeneous preparations. The final detergent and aqueous phases were precipitated in 50% acetone (vol/vol) at −20°C overnight, and material was solubilized in 3× Laemmli buffer for subsequent immunoblot analyses.

### Invasion assay.

HeLa229 cells were prepared in 24-well plates with 12 mm coverslips, and invasion assays were performed essentially as described (9). Density gradient purified EBs were used at a multiplicity of infection (MOI) of 20. Where appropriate, host cells were treated with medium containing 100 µM EIPA, 20 µM casin, 25 µM wiskostatin, 25 µM Ehop-016, or 200 µM CK666 (all purchased from Sigma-Aldrich) for 15 min prior to infection. Cultures were either mock treated or maintained with inhibitors during infection and subsequent incubation. Infections were done on ice with rocking for 1 h and then shifted to 37°C for 45 min. The cultures were fixed with 4% paraformaldehyde, and extracellular or intracellular EBs were differentially labeled with murine LPS-specific or rabbit MOMP antibodies, respectively. Detection was accomplished with secondary antibody conjugated to Alexa-594 (anti-mouse) or Alexa-488 (anti-rabbit). Percentages of invaded chlamydiae were computed by enumeration of internal and external chlamydiae in 10 fields of view. The percentage EB internalization was calculated via the formula ([total EBs – external EBs]/total red EBs) × 100 = percent (%) invasion.

### Proximity labeling.

BirA-mediated biotinylation of host proteins was accomplished according to established protocols (68). HeLa cells were nucleofected with empty pcDNA3.1 mycBioID as a BirA-only control or with TmeA- and TmeB-containing constructs. For control experiments, parallel cultures were cultivated in RMPI supplemented with dialyzed FBS with or without 50 µM biotin. For proximity labeling studies, cultures were maintained for 24 h prior to harvest in RPMI supplemented with 10% FBS and 50 µM biotin. Proximity labeling with APEX2 was also performed as described (39). Briefly, HeLa cells were cultivated in one 6-well plate per experimental condition and Dulbecco modified Eagle medium (DMEM) + 10% FBS. Once confluent, monolayers were either mock infected or infected with WT, TmeA-APEX, or TmeB-APEX by spinning at 900 × *g* for 60 min to reach an MOI of ca. 2. EBs used for infection were previously cultivated in the presence of anhydrotetracycline (ATc) for expression of TmeA-APEX and TmeB-APEX prior to invasion. After incubating at 37°C for 24 h in growth medium supplemented with 50 ng/ml ATc, 1.5 mM final concentration Biotin-phenol was added to medium and incubated for 30 min. Biotinylation was catalyzed by replacing the medium with 3 mM H_2_O_2_ in phosphate-buffered saline (PBS) for 1 min and then washing cultures 3 times with quenching solution. For both BirA and APEX approaches, cultures were harvested into RIPA buffer (50 mM Tris, pH 7.4, 150 mM NaCl, 1% NP-40, 0.5% Na deoxycholate, 0.1% SDS) supplemented with protease inhibitors and incubated on ice for 1 h. The insoluble fraction was separated by spinning at 17,000 × *g* for 3 min, and then the soluble fraction was applied to equilibrated high-capacity NeutrAvidin Agarose (Thermo Scientific) and rocked overnight at 4°C. The resin was washed 3 times, and biotinylated proteins were eluted in 40 µl 3× Laemmli buffer at 95°C for 4 min. For identification of biotinylated proteins using mass spectrometry, proteins were run into a 12% SDS-PAGE gel for 15 min at 200 V and then stained with Sypro Ruby protein blot stain (Lonza) and cut into lanes. The University of Kentucky Proteomics Core performed digestion, preparation, and analysis of samples using Mascot data analysis software. A TSQ Vantage triple quadrupole mass spectrometer was used for liquid chromatography with tandem mass spectrometry (LC-MS/MS) protein identification.

### Immunoprecipitation.

HeLa 229 cells nucleofected to ectopically express TmeA-FT, TmeB-FT, or Cpn0678-FT were seeded in one 6-well plate each. Then, 24 h postnucleofection, cultures were harvested in NP-40 buffer (69) and incubated on ice for 1 h. Insoluble material was separated by spinning at 17,000 × *g* for 3 min at 4°C. Soluble fractions were precleared with equilibrated Sepharose 4B (Sigma) for 1 h with rocking at 4°C and then applied to equilibrated anti-Flag M2 affinity gel (Sigma) overnight. The resin was washed 3 times with lysis buffer, and FT proteins were eluted with 3× Flag peptide in PBS. Then, 6× Laemmli buffer was added to eluates prior to separation on SDS-PAGE gels for immunoblotting.

### Pyrene assay.

Pyrene actin polymerization assays were performed as previously described (24). Briefly, monomeric pyrene-labeled actin was prepared by diluting lyophilized pyrene actin (cytoskeleton) in 5 mM Tris (pH 8.0) 0.2 mM CaCl_2_ 0.2 mM ATP (G buffer) and incubating on ice for 1 h. Monomeric pyrene actin was obtained by collecting the supernatant after a 90-min, 100,000 × *g*, 4°C spin in a Beckman Optima MAX TL ultracentrifuge using a TLA 55 rotor (Beckman Coulter). N-WASP ΔEVH1 was employed as previously reported to facilitate purification from E. coli (70). Approximately 30 µg of pyrene-labeled actin was mixed with 1 to 2 µg of the indicated proteins (TmeA, N-WASP^151-501^, Arp2/3, TarP) in a volume of 500 µl for 5 min before the addition of 1/20th volume of polymerization buffer (500 mM KCl, 20 mM MgCl_2_, 10 mM ATP). The reaction (contained in a semimicrocuvette and holder assembly) was monitored for 30 min with an LS 55 luminescence spectrophotometer equipped with the biokinetic accessory and directed by FL Winlab software version 4.0 (Perkin-Elmer, Beaconsfield, Bucks, United Kingdom) with 2.5-nm bandwidth at 365-nm excitation wavelength and a 2.5-nm bandwidth at 407-nm emission wavelength.

### Statistical analysis.

Unless otherwise noted, the presented data are representative from triplicate experiments where quantitative data were generated from experiments containing triplicate biological replicates. Calculation of the standard deviation of the mean and assessment via Student’s *t* test statistical analyses were performed using GraphPad Prism 6 version 6.04 (GraphPad Software, Inc.).
