# Supplementary figures and images for: Chlamydia trachomatis TmeA Directly Activates N-WASP To Promote Actin Polymerization and Functions Synergistically with TarP during Invasion
Source: mBio. 2021 Jan 19;12(1):e02861-20. doi: 10.1128/mBio.02861-20 (PMC7845632; doi:10.1128/mBio.02861-20)

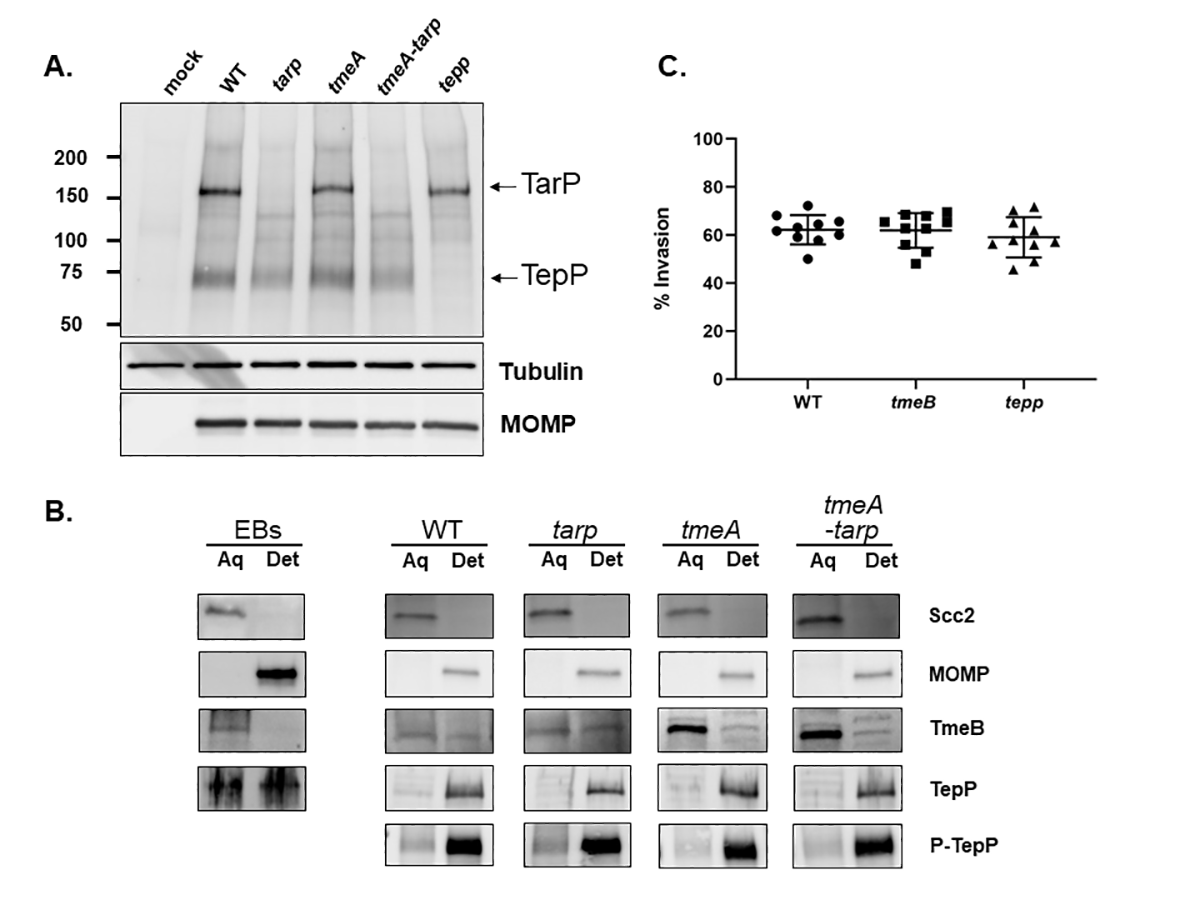

Supplement: FIG S1 [file mBio.02861-20-sf001.tif]

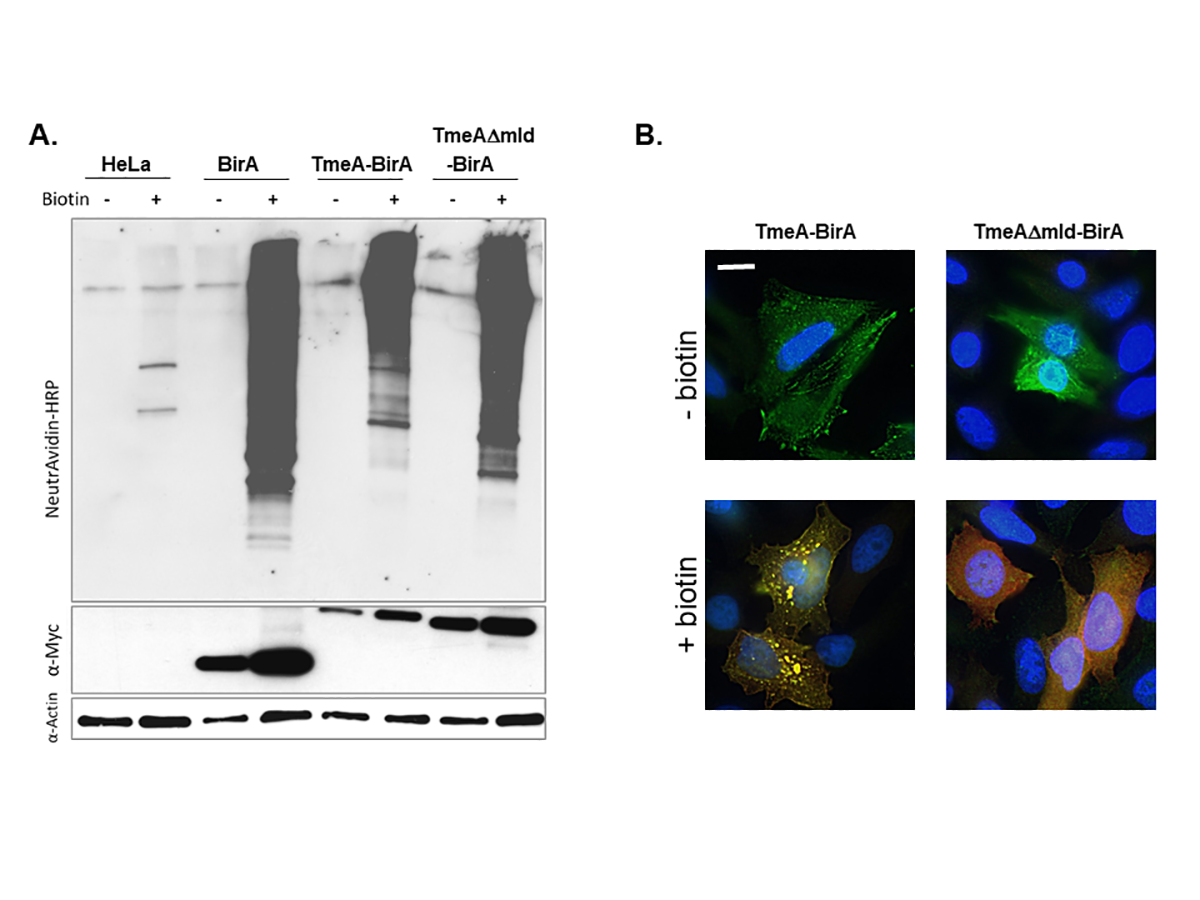

Supplement: FIG S2 [file mBio.02861-20-sf002.tif]

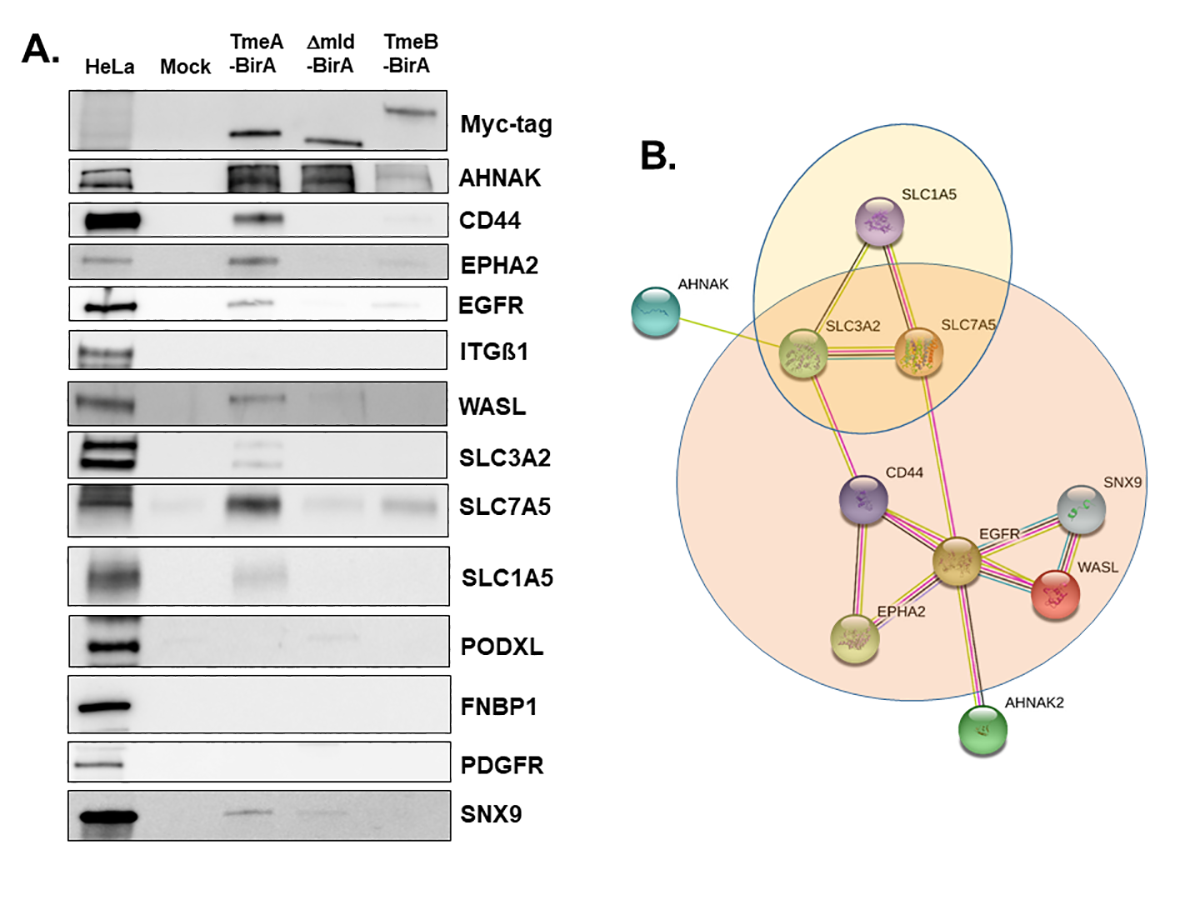

Supplement: FIG S3 [file mBio.02861-20-sf003.tif]

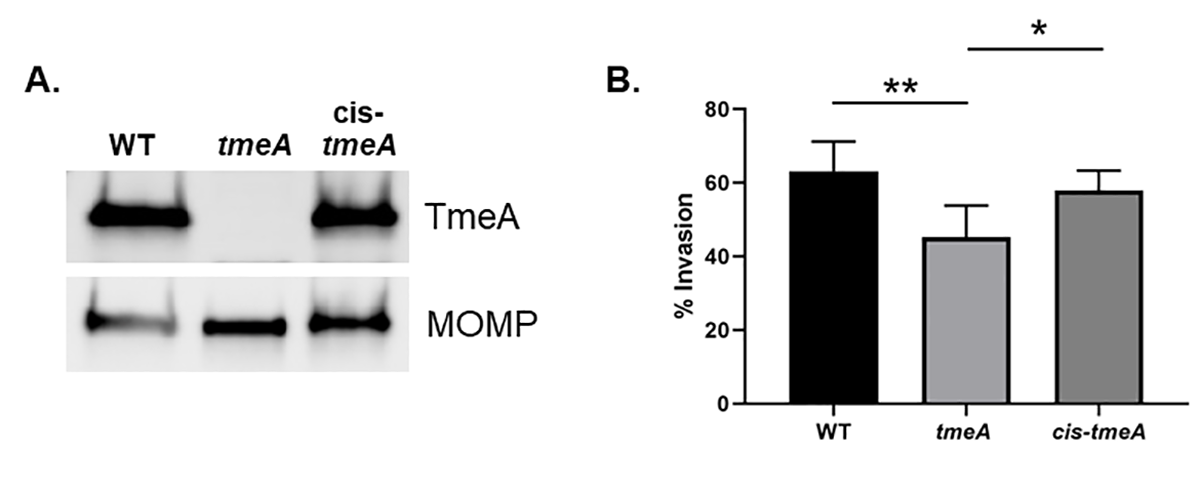

Supplement: FIG S4 [file mBio.02861-20-sf004.tif]

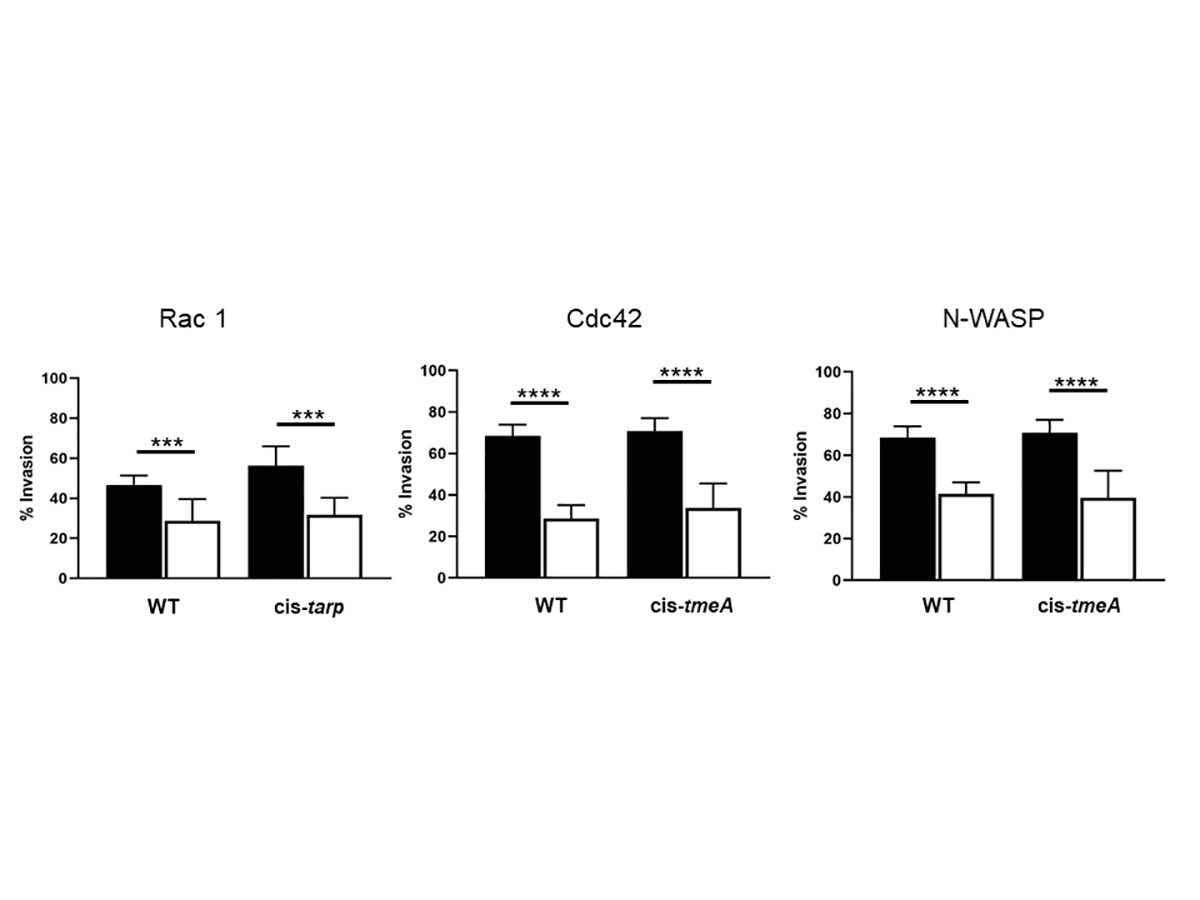

Supplement: FIG S5 [file mBio.02861-20-sf005.tif]
